# A Web-Based Resource Informed by Cognitive Behavioral Therapy and Positive Psychology to Address Stress, Negative Affect, and Problematic Alcohol Use: A Usability and Descriptive Study

**DOI:** 10.2196/63819

**Published:** 2025-02-10

**Authors:** Ingrid Serck-Hanssen, Marit Solheim-Witt, Justin J Anker, Dawn E Sugarman

**Affiliations:** 1 Department of Clinical Counseling, Alpha Element Institute, LLC, 6160 Summit Dr N, Suite 200, Minneapolis, MN, 55430, United States, 1 6123093659; 2 Department of Holistic Health, Alpha Element Institute, LLC, Minneapolis, MN, United States; 3 Psychiatry and Behavioral Sciences, University of Minnesota, Minneapolis, MN, United States; 4 Division of Alcohol, Drugs, and Addiction, McLean Hospital, Belmont, MA, United States; 5 Department of Psychiatry, Harvard Medical School, Boston, MA, United States

**Keywords:** alcohol misuse, stress, drinking to cope, DTC, negative affect, positive psychology intervention, PPI, cognitive behavior therapy, CBT, alcohol use, drinking, usability, descriptive study, behavior, emotion, coping skill, positive psychology, psychology, online resource, mobile phone

## Abstract

**Background:**

Research documents that drinking to cope behavior can be disrupted by enhancing emotion regulation and coping skills related to the experience of stress and negative affect. The Alpha Element Self-Coaching Plan incorporates principles of positive psychology and cognitive behavioral therapy to redirect negative thinking and emotions and, therefore, has the potential to benefit individuals who use alcohol to cope with stress.

**Objective:**

This study aimed to evaluate satisfaction and usability of the web-based Alpha Element Self-Coaching Plan in order to inform the development of an expanded digital platform based on the Alpha Element framework.

**Methods:**

Participants enrolled in the web-based program as part of their clinical care were eligible to participate. A total of 20 individuals (14 women and 6 men) between ages 30 and 79 (mean 54.5, SD 14.14) years completed web-based questionnaires to assess product performance in areas such as ease of technology use, quality of videos and handouts, and the value of the activities. Participants also completed the System Usability Scale (SUS) and provided background and demographic information, including alcohol use.

**Results:**

Only 1 participant reported no alcohol use in the past year; 55% (11/20) of participants drank alcohol 2‐4 times per month or less and 45% (9/20) reported drinking alcohol 2‐3 times per week or more. The average SUS score of 76.38 (SD 17.85) was well above the commonly accepted threshold of 68, indicating high system usability. A majority of the sample (16/19, 84%) agreed or strongly agreed that the activities in the program inspired behavioral changes; and most agreed or strongly agreed that the program was engaging (16/20, 80%), well-organized (18/20, 90%), and easy to follow (17/20, 85%). Only 2 participants endorsed experiencing difficulty using the program on a smartphone. Suggestions for program improvements included expanding the platform, updating the web format, adding user interactivity, and enhancing navigation.

**Conclusions:**

These data suggest that participants were generally satisfied with the web-based Alpha Element Self-Coaching Plan, and rated usability of the program as favorable. Importantly, a significant portion of participants reported that the program inspired behavioral changes. More research is needed with a larger sample to obtain specific data about alcohol consumption and investigate associations between alcohol use and program components, as well as examine gender differences. Data collected from this study will be used to expand the platform and improve user experience.

## Introduction

Stress is a well-documented risk factor associated with the initiation, maintenance and relapse of alcohol use disorder [1]. The self-medication model suggests some individuals drink to cope (DTC) with stress due to the belief that drinking will alleviate stress and negative affect [2,3]. However, although alcohol may provide temporary relief from emotional discomfort, in the long-term, drinking to cope with stress exacerbates negative affect and puts individuals at risk for alcohol-related consequences [4].

Positive psychology interventions (PPIs) may be beneficial in disrupting the DTC cycle of alcohol use, as emerging data suggest that the processes underlying PPI may facilitate more adaptive behavioral choices about substance use [5]. PPIs are strength-based approaches that apply individual traits along with positive subjective experiences to promote well-being [5,6]. PPIs aim to foster optimism, psychological well-being, and resilience through practices like cognitive flexibility, positive reframing, and the cultivation of strengths, representing a shift away from focusing solely on reducing symptoms and instead building emotional assets. Furthermore, research indicates that positivity can be learned and lead to increased well-being and decreased alcohol consumption [7]. A wide range of PPIs exist that vary in type, delivery mode, and intensity. PPIs have mostly been studied in nonclinical samples; however, preliminary evidence exists for their efficacy in clinical settings [8]. Research suggests that cognitive changes may explain the positive effect of PPIs on emotions [6]. Therefore, cognitive behavioral therapy (CBT) can be personalized with PPI components that target motivational and emotional factors. With a focus on increasing positive emotions, behaviors, and cognitions, PPI approaches align well with CBT principles and both can be leveraged with the objective to reduce stress with negative affect aimed at mitigating cravings for alcohol to self-medicate.

Critical to the success of CBT-based strategies in the clinical setting is engaging clients in homework exercises [9,10]. Technology-based tools such as smartphone apps and web-based self-management programs provide scalable solutions for individuals to engage with self-directed exercises. Emerging research indicates that web-based interventions can serve a protective function for mental health by mitigating the effects of stress [11]. Ongoing measurement of engagement with web-based homework exercises along with strategic approaches to tailoring intervention content delivery to individuals may help increase sustained practice [12]. The purpose of this quality improvement (QI) study was to evaluate usability and satisfaction of the web-based Alpha Element Self-Coaching Plan (hereafter referred to as the Self-Coaching Plan [13]) applied in a community clinical counseling setting. The Self-Coaching Plan incorporates a combination of CBT and PPI approaches and features content based on a novel positive psychological framework of Vitality Type patterns in behavior and cognition [14-17]. The goal of the Self-Coaching Plan is to empower individuals with tools to discover a new and positive perspective on themselves, along with psychoeducation and personalized skills to proactively manage stress and negative affect, as well as address problematic drinking behavior. The aim of this QI study was to examine satisfaction and usability of the web-based Self-Coaching Plan in order to facilitate ongoing improvement of this tool.

## Methods

### Study Setting

In this QI study the Self-Coaching Plan was offered as adjunct homework to clients in the clinical setting. Participation in the project was optional and did not affect the therapeutic relationship with their clinical provider. All participants in this study were receiving mental health counseling through telehealth in a community counseling clinic in Minneapolis, Minnesota.

### Study Design

Data were gathered for administrative purposes within the context of routine efforts to examine and improve clinical care services and operations. This QI study was conceptualized as a formative evaluation to assess product performance of the Self-Coaching Plan. The evaluation surveys focused on system usability, technology ease of use, quality of videos, and downloadable handouts, as well as the value of the program content and activities.

### Participants

Participants were recruited through a self-selection process. We applied an all-comers approach, meaning individuals enrolled in the Self-Coaching Plan as part of their standard care were invited to participate. Of the 35 clients invited to participate, 20 (14 women and 6 men) agreed. To recruit participants, a University of Minnesota REDCap (Research Electronic Data Capture; Vanderbilt University) survey link was included in weekly touch-base emails.

### Intervention

A web-based tool for self-directed learning, the Self-Coaching Plan, was applied in this study as a homework resource in the clinical setting. The Self-Coaching Plan is based on the premise that introducing individuals to a framework of 4 Vitality Type patterns as a strategy to disrupt negative self-schemas can help provide new perspectives on existing behavior patterns as well as cognitive resources to reframe distorted thinking that is impairing the regulation of negative affect (Table 1).

**Table 1.** The premise of the Self-Coaching Plan is to apply 4 Vitality Type categories to self-identify empowered and disempowered patterns in behavior and cognition. Throughout the Self-Coaching Plan, individuals are reminded that words are cognitive tools that frame our perceptions and shape our reality.

Vitality Type patterns	Catalyst 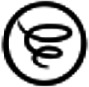	Torchbearer 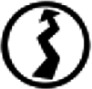	Pathfinder 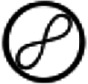	Pragmatist 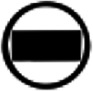
Empowered behavior patterns	Discovery Open-minded Uplifting Playful Social “Let’s try it now.”	Action Direct Fast Results-driven Bold “What can I do?”	Development Investigative Process-oriented Diplomatic Consensus-building “I’ll think about it.”	Completion Scrutinizing Focused Discerning Structured “It has to be done right.”
Disempowered behavior patterns	Self-indulgence Erratic	Irritability Restless	Avoidance Overwhelmed	Perfectionism Judgmental

The 4 categories, Catalyst Vitality, Torchbearer Vitality, Pathfinder Vitality, and Pragmatist Vitality, represent distinct patterns in outlook and behavior (Table 1). The essence of a catalyst is to spark activity. Positive words associated with the Catalyst Vitality pattern include discovery, open-minded, uplifting, playful, and social. The essence of a torch bearer is to challenge status quo and pave the way to take action. Positive words associated with the Torchbearer Vitality pattern are action, direct, fast, results-driven, and bold. The essence of a pathfinder is to be methodical. Positive words associated with the Pathfinder Vitality pattern include development, investigative, process-oriented, diplomatic, and consensus-building. The essence of a pragmatist is to bring clarity and stability to their environment. Positive words associated with the Pragmatist Vitality pattern are completion, scrutinizing, focused, discerning, and structured. As part of the Self-Coaching Plan, participants study all 4 Vitality Type categories by exploring the words linked to each. Through a series of exercises, they apply these words to personalize their self-coaching strategies. By organizing positive words into categories (Table 1) the Vitality Type patterns combine elements from both CBT and PPI.

Exploring how to break unhealthy habits and cognitive distortions and replacing them with positive self-talk can create shifts in outlook that can lead to changes in behavior. The Vitality Type categories represent a range of possibilities in patterns of cognition and behavior which in the Self-Coaching Plan are applied as cognitive tools to help individuals self-identify and articulate aspects of their empowered and disempowered patterns. Exploring words linked to the Vitality Type patterns and applying these words as cognitive tools to change behavior and outlook, help individuals take back their power instead of leaning on alcohol and other negative coping strategies. It is posited that the framework of 4 Vitality Type categories offers resources to promote positive self-perception to inspire new self-awareness, self-discovery, and development of personalized self-management strategies. Vitality Type patterns can also be applied as a cognitive strategy for considering differences in relationships and interpersonal communication. Managing relationships with self and others are key aspects of managing stress and problematic drinking behavior.

It is proposed that learning about these patterns (schemas) can help individuals orient and learn new terminology to reframe negative schemas they have about themselves. By applying the constructs of 4 Vitality Types as cognitive resources to identify and articulate empowered and disempowered patterns, individuals are introduced to a fresh perspective on themselves along with terminology that can provide a bridge to describe potential movement away from pathology toward hope and well-being. This self-reflective process can lead to a deeper understanding of how behaviors and thoughts impact outlook and overall health. By using the Vitality Type categories as a framework of schemas and tools to foster cognitive flexibility, the Self-Coaching Plan adapts CBT elements and PPI principles as a therapeutic strategy to create synergy and inspire self-investigation. Therefore, the terminology of the 4 Vitality Type categories are applied to all activities to provide a map for organizing and personalizing CBT-PPI strategies.

In this QI study, the objective of the Self-Coaching Plan was for clients, between sessions, to explore and process patterns of disempowerment and negative outlook and learn skills to redirect and self-regulate. This web-based program offers psychoeducation and activities through videos and visual graphics; topics include how to apply the Vitality Type patterns to foster self-compassion, growth mindset, and preparing for positive change as well as facts about gender-specific vulnerabilities and substance use. During the Self-Coaching Plan, individuals are encouraged to use the Vitality Type patterns as tools to help achieve their goals and focus on what they are for—as a shift away from focusing on what they are against and working to change. Self-Coaching Plan activities are organized into sections with the objective of completing an action plan to stay motivated on their journey toward positive change (Table 2). It is recommended that the proposed order of activities is followed, however, individuals are free to choose the order they prefer. The goal is for individuals to apply the Vitality Type framework to gain a new perspective on predominant go-to behavior patterns and understand the importance of developing their less dominant patterns to self-advocate, set boundaries, and feel more empowered. The Self-Coaching Plan is designed to help individuals get prepared and stay motivated to achieve their goals over an 8-week period. They learn to apply words as cognitive tools to change their behavior and take their power back instead of leaning on alcohol and other negative coping strategies. The Self-Coaching Plan can be applied as both a guided and unguided protocol.

**Table 2.** A web-based tool for self-directed learning, the Self-Coaching Plan, was applied in this study as a homework resource in the clinical setting. The goal was to empower individuals through a personalized process of activities and self-reflection to foster healthy habits, learn to set boundaries, manage emotions, and change unhealthy habits such as problematic drinking behavior.

Module name	Description	Components
Growth Mindset	Psychoeducation about how our abilities and frame of mind can be developed through dedication and hard work. A growth mindset is knowing that it is never too late to make a change.	Welcome video. Course intro. Psychoeducation about growth mindset and self-compassion.
Get Ready for Positive Change	Psychoeducation to get clarity about priorities and steps to achieve positive lifestyle changes.	Course objectives. Gender specific vulnerabilities for substance use. Substance use and drug facts.
Vitality Type Indicator	The objective of this self-assessment is to gain awareness of empowered and disempowered patterns in communication and relationships.	Self-assessment objective. Connection between stress management, outlook, and drinking behavior.
Vitality Type Patterns	Psychoeducation about how managing the relationship with self and others is key to managing the relationship with alcohol.	Introducing the 4 Vitality Types as tools to self-manage and lay the foundation for developing a growth mindset. Vitality Type matrix of empowered and disempowered patterns.
Top Priority	Identify the most important area of desired change. Individuals are encouraged to look at themselves as a priority.	Exploring boundaries required for positive change. Self-care as a means to manage stress. Connection between mind, body and soul in healing.
Self-Coaching Guide	The Self-Coaching Guide is the Vitality Type of the highest score on the Vitality Type Indicator self-assessment.	The approach of the Self-Coaching Guide is key to achieving our Top Priority. Self-Coaching exercise applies the highest scoring Vitality Type pattern as a technique and practice to reflect on goals, challenges, and personal growth. Exercise to create positive “I am” statements using words from the word-bank associated with their highest self-assessed Vitality Type score.
Hidden Resource	The Hidden Resource is the Vitality Type of the lowest score on the Vitality Type Indicator self-assessment.	The approach of the Hidden Resource indicates untapped potential that can help on the path to sobriety. Exercise to redirect thoughts by applying the lowest scoring Vitality Type as a strategy to address challenges and difficult situations in a new way. Reflection exercise to discover how to leverage aspects of the Hidden Resource approach as support during the healing journey.
Shadow Aspects	Shadow Aspects represent disempowered behaviors taken on during times of stress or fear.	Exercise to self-identify behaviors that are barriers in communication and relationships. Reflection activity about self-compassion and growth mindset as strategies to help turn around a disempowered state.
Power Words	Power Words are positive characteristics and behaviors linked to each Vitality Type.	Activities to choose Power Words, which are then applied to personalize strategies to help disrupt Shadow Aspects. Exercise to identify 1 Power Word linked to the Self-Coaching Guide and another word linked to the Hidden Resource as strategy to stay proactive rather than reactive in stressful situations. Reflection activity to become more aware of how to deal with joy and conflict can be key to understanding the relationship with alcohol.
Self-Coaching Plan Commitment Sheet	Course summary: maintaining sobriety and a sober-minded lifestyle is much more manageable when the focus is on empowerment, growth mindset and self-compassion.	Resources to explore how to continue using the Vitality Types to stay committed to healing, health and wellness.

As part of the Self-Coaching Plan, individuals take a self-assessment (Vitality Type Indicator, Table 2) that sorts responses according to the categories of the 4 Vitality Type categories. The results of the self-assessment provide a map for individuals to discover a range of Vitality Type patterns in their behavior, cognition, and communication. Individuals’ highest Vitality Type score indicates a predominant pattern and is identified as their Self-Coaching Guide. An individual’s lowest Vitality Type score indicates untapped potential and is identified as their Hidden Resource. Attributes linked to an individual’s Hidden Resource represent capabilities outside of the person’s typical patterns of behavior. The concepts of Self-Coaching Guide and Hidden Resource are incorporated into the program as cognitive tools and a positive approach to develop personalized strategies for stress-management and self-regulation. Individuals are prompted to practice their Hidden Resource capabilities in order to prepare themselves to proactively handle challenges and difficult situations with a new perspective. This information is applied in the Self-Coaching Plan as CBT-PPI informed reflective learning activities and homework assignments.

Through a series of activities of using words as cognitive resources to make positive shifts, individuals practice using specific words that represent empowered behavior to formulate their desired change and develop healthy habits. A word bank is linked to each of the 4 Vitality Type categories as a tool to discover a range of possibilities. Individuals are asked to reflect on which words and aspects of the 4 patterns resonate with their view of themselves, and which do not. In a self-coaching module, individuals explore how to use their Self-Coaching Guide and their Hidden Resource (Table 2) to manage stress and move toward sobriety and a healthier, more balanced life. Table 1 presents an overview of the 4 categories and illustrates how each proactive (empowered) Vitality Type pattern has a reactive (disempowered) opposite. In the Self-Coaching Plan (Table 2), the disempowered opposite is referred to as a Shadow Aspect. Shadow Aspects represent disempowered behaviors taken on during times of stress or fear. To dig deeper into understanding their disempowered reactions, participants are asked to self-identify 2 primary Shadow Aspects. To redirect disempowerment, individuals then choose 2 words from the word bank they consider to be empowering. These words are referred to as Power Words. Power Words are positive characteristics and behaviors linked to each Vitality Type which are applied to develop their personalized strategies to redirect. During this CBT-PPI process of applying Vitality Type categories in self-discovery and reflective learning, individuals gain a newfound perspective on their go-to behavior patterns along with personalized strategies to use their less dominant patterns as a resource to set boundaries, self-advocate, and feel more empowered.

### Procedures

A survey was offered through a REDCap link as part of touch-base emails frequently sent out to all registered users of the Self-Coaching Plan. We informed all users that the goal of the project was quality improvement and their answers would be used to improve user experience. Users were not able to proceed to the survey unless they had checked the box indicating they agreed to participate. Participants completed a custom questionnaire, the System Usability Scale (SUS), as well as background and demographic information, including alcohol use. The custom questionnaire was designed to evaluate (1) the relevance and ease-of use of the Self-Coaching Plan material and (2) satisfaction with the Self-Coaching Plan.

### Measures

The SUS is a 10-item scale designed to provide a global view of subjective assessments of usability. The scale covers a variety of aspects of usability, including the need for support and training while using the system as well as its level of complexity. The SUS evaluation instrument yields a single number that represents a composite measure of the overall usability of the system being studied with current literature suggesting that a score of 68 as a useful benchmark (mean SUS score) [18].

The custom questionnaire was developed to gather feedback about program engagement along with dimensions of perceived interactivity and ease of use. The questions were developed by creators of the Self-Coaching Plan and other subject matter experts. The data were collected to establish a baseline along key dimensions against which change over time can be measured. Key dimensions measured included strengths and limitations of the technology, videos, and exercises; need for tech support, notifications, and reminders; program navigation and logistics features; time commitment; aesthetics and visual elements; as well as suggestions for improvement. Specific questions were asked about engagement, self-perception and behavior change as well as knowledge about key terms and concepts, such as Vitality Types, Self-Coaching Guide, and Hidden Resource. Each item was rated on a 5-point Likert scale ranging from strongly disagree to strongly agree with a midpoint of neither agree nor disagree. Recommendations for enhancing program improvement were obtained through optional write-in responses to capture comments and suggestions in the language of participants. Alcohol consumption was measured with 1 question about frequency of alcohol use in the past year with the following scale: Never; Monthly or less; 2‐4 times a month; 2‐3 times a week; 4 or more times.

### Data Collection

Participants completed the surveys through REDCap. REDCap is an encrypted, electronic database that is HIPAA (Health Insurance Portability and Accountability Act) compliant. It is approved by University of Minnesota Institutional Review Board for the administration and storage of human subject information.

### Statistical Analysis

Analyses for this study focused on evaluating the Self-Coaching Plan using descriptive statistics and were performed using R statistical software (R Foundation for Statistical Computing). Mean and standard deviation were used for continuous variables such as age and SUS scores. For categorical variables, including demographic information (eg, sex, education level, and race) and survey responses (eg, engagement with self-care routine and frequency of alcohol consumption), frequencies were calculated.

### Ethical Considerations

The project was determined not to qualify as human subject research by the University of Minnesota Institutional Review Board (STUDY00020268). Confidentiality was strictly protected. No identifying information was collected.

## Results

### Participant Characteristics

Participants (N=20) were between 30 and 79 years of age (mean 54.5, SD 14.14), predominantly female (n=14, 70%) White (n=15, 75%) and well educated with 35% (n=7) college graduates and 35% (n=7) reporting as graduate level or above. Only 1 participant reported no alcohol use in the past year; 55% (n=11) of the sample drank alcohol 2‐4 times per month or less and 45% (n=9) reported drinking alcohol 2‐3 times per week or more.

### System Usability

Average SUS score from this study was 76.38 (SD 17.85). This result is well above the commonly accepted threshold of 68. The score indicates high usability of the system and its interfaces.

### Custom Questionnaire

As shown in Table 3, a majority (16/19, 84%) of participants agreed or strongly agreed that the activities in the program inspired positive behavioral changes while 90% (18/20) agreed or strongly agreed that participation in the program positively influenced their self-perception. There was strong endorsement of knowledge and understanding about concepts unique to the program such as Vitality Types, Self-Coaching Guide and Hidden Resource. Of note, all participants (n=20, 100%) agreed or strongly agreed that the Vitality Type Indicator self-assessment was a helpful tool in their self-development; 90% (n=18) endorsed the purpose of the Self-Coaching Guide as clear and understandable while 85% (n=17) reported they could relate to their Self-Coaching Guide; 80% (n=16) of participants strongly endorsed understanding the concept of a Hidden Resource. A majority of participants (n=16, 80%) endorsed the program as engaging and 80% (n=16) found the videos were effective in motivating them to complete the homework activities and most participants agreed or strongly agreed that the visual design of the platform was appealing. With regards to technical interface, 80% (n=16) of participants did not experience significant difficulties using the Self-Coaching Plan on smartphones or computers. Generally, participants (n=18, 90%) found the program well-organized.

**Table 3.** Results from the quality improvement study (N=20).

Quality improvement survey questions	Strongly agreed or agreed, n (%)	Neither agreed or disagreed, n (%)	Disagreed or strongly disagreed, n (%)
The activities in this program inspired me to change my behavior.^a^	16 (84)	—^b^	3 (16)
Participating in this course positively impacted my self-perception.	18 (90)	2 (10)	—
The order of the activities in the program was logical and helped in my understanding.	18 (90)	1 (5)	1 (5)
The videos were effective in motivating me to complete the activities.	16 (80)	3 (15)	1 (5)
The Vitality Type categories were understandable.	18 (90)	2 (10)	—
The Vitality Type Indicator self-assessment was helpful in my self-development.	20 (100)	—	—
The purpose of the Self-Coaching Guide was clearly explained and understandable.	18 (90)	2 (10)	—
I related to my Self-Coaching Guide.	17 (85)	3 (15)	—
The concept of a Hidden Resource was clear to me.	16 (80)	4 (20)	—
I found the program to be engaging.	16 (80)	2 (10)	2 (10)
The content within the program was well organized.	18 (90)	2 (10)	—
The time required to complete this course felt appropriate to me.	17 (85)	2 (10)	1 (5)

a One participant missed this item.

b Not endorsed by any participants.

As part of the custom questionnaire, participants were asked to respond to 3 open-ended questions. The first question asked participants to share their thoughts about which program components they considered to be the most effective. Predominant themes that emerged from responses to this question were (1) empowerment, (2) personalization, and (3) engagement. The following comments illustrate a sense of empowerment: “Understanding better how I react to things” and “A clear view of my strengths and weaknesses really helped me choose where I wanted to focus my energy.” The theme personalization was illustrated by the following quotes: “I love that I get to use a personal Self-Coaching Guide based on my behaviors specifically designed for me!” and “I enjoyed exploring the Vitality Types that align with my personality and observing how they influence my daily behavior. After completing the test (Vitality Type self-assessment), I identified which trait was most prominent for me.” Third, the theme engagement, was illustrated by these comments: “I found the homework was very helpful for me to have. It encouraged me to continue my healing in between sessions and then to have a follow-up conversation with my therapist about the homework really helped tie everything together for me.” “The activities were most effective because they made you actually pause and reflect.”

The second open-ended question asked participants to share their views about program components they considered the least effective. Here the predominant theme was navigation, as illustrated by the following comments, “I did find it a little confusing in the beginning to find the correct files at the end of the program,” and “It was hard to find the Vitality Types breakdowns through the library.” The third open-ended question asked for recommendations to enhance the effectiveness of the program. Here feedback again focused on navigation, as the following comments illustrate. “I think the navigation could be cleaned up, but overall, it was an effective program.” “The online interface needs to be updated.” Other themes for program improvements included expanding the platform and adding user interactivity as illustrated by the following comments: “I’d be excited to witness user interaction on the platform. In addition, incorporating a gamification element, perhaps tracking our progress through the program, would be an engaging feature.” Another participant stated, “I hope for a platform where I can engage with others regarding Vitality Types, perhaps in the form of a community for individuals who have undergone this course.”

## Discussion

### Principal Findings

Findings from this study document high usability and satisfaction with the web-based Self-Coaching Plan in the clinical setting. A significant portion of participants reported that the program inspired behavioral changes as well as positive self-perception and found the program to be well-organized and easy to follow. Most participants (18/20, 90%) reported the Vitality Type categories were understandable, with 85% (17/20) stating they could relate to their Self-Coaching Guide, and 84% (16/19) endorsed that they were inspired by program activities to change their behavior. However, more research is needed to examine the relationship between these constructs, participant self-perception, and behavior change regarding alcohol use. Several participants expressed a desire for improved navigation.

### Comparison With Previous Work

An increasing amount of research points to the double jeopardy of stress and negative affect as a detrimental set of vulnerabilities that lead to problematic drinking behavior and DTC [2,19]. This trend is of increasing concern and spotlights the need for research and development of innovative self-management approaches. Data from this QI study suggest that participants were generally satisfied with the Self-Coaching Plan, and rated usability of the program as favorable. The implications from this study support the application of the Vitality Type categories as a CBT-PPI approach for individuals to gain a fresh and more positive self-perception as well as personalized skills to self-regulate. All participants endorsed the Vitality Type Indicator as a helpful tool in their self-development. Through reflection activities, individuals were encouraged to discover a new view of themselves and apply the Vitality Type patterns as cognitive tools to disrupt barriers, reframe, and manage stress.

Findings from this QI study indicate the 4 Vitality Types patterns can be adapted to a web-based program for individuals to orient and reflect on these 4 categories of attributes and explore the possibility of using the terminology to describe aspects of themselves and develop strategies to redirect. Responses to the custom questionnaire document that 90% (18/20) of participants endorsed the purpose of the Self-Coaching Guide as clear and understandable. Furthermore, 85% (17/20) reported they could relate to their Self-Coaching Guide. The majority of participants 80% (16/20) strongly endorsed understanding the concept of a Hidden Resource. It is noteworthy that 90% (18/20) of participants thought the Self-Coaching Plan positively impacted their self-perception.

A focus of CBT approaches is to support clients in their efforts to examine their thoughts and behaviors and become scientific investigators of their own lives [9]. Results from the open-ended questions in the custom questionnaire document that participants applied the self-directed exercises in the Self-Coaching Plan as tools to self-reflect and personalize the content. This finding is underscored by the comment, “The activities were most effective because they made you actually pause and reflect.” Individuals who engage with homework activities are taking a hopeful step by demonstrating the belief that their own efforts will help them overcome their clinical problems [10]. It has been proposed that integrating PPIs into existing treatment approaches may be beneficial because they produce an immediate reward that is nonsubstance-related [20].

Historically, PPI research has focused on individual positive qualities and attributes such as gratitude, optimism, and kindness [21] and not development of novel conceptual frameworks of patterns in behavior and cognition to address the larger context of disempowerment created by negative self-schemas. The Vitality Type categories offer a map of words for individuals to reflect on a range of factors impacting their behavior and communication without focusing on pathology. The Self-Coaching Plan is distinguished from other web-based psychosocial programs by providing a novel conceptual framework of 4 Vitality Type categories as proposed schemas in behavior and cognition. The proposed schematic conceptualizations of distinct patterns of emotion, behavior and cognitions as 4 Vitality Type patterns represent a model that can be applied to expand upon the traditional positive psychology approaches. As a psychosocial approach to self-development, the Self-Coaching Plan represents a new context for self-reflection and an innovative way of looking at differences in relationships and interpersonal communication. The current lack of standardized PPI protocols may limit the implementation of PPIs in the clinical setting and it may be beneficial to develop new, positive-focused interventions for substance use and other conditions instead of simply including traditional PPIs into existing treatments [22]. One study found that addiction counselors were using positive psychology exercises in their sessions over half of the time without classifying their methods as explicitly PPI [23]. To our knowledge, current web-based PPI informed programs do not apply a map of 4 empowered and disempowered patterns in behavior and cognition as schemas to navigate stress and proactively manage negative affect. As documented by the findings in this study, 90% (18/20) of participants endorsed the Self-Coaching Plan as positively impacting their self-perception. These results indicate that awareness of the Vitality Type categories and terminology can be leveraged to help individuals gain a more optimistic view of themselves as well as develop a personalized self-coaching plan to work toward health and well-being.

Data collected from this study will be applied to improve and expand the web-based Self-Coaching Plan. More research is needed with a larger sample to obtain specific data about alcohol consumption and investigate associations between alcohol use and program components. Furthermore, web-based programs that incorporate gender-specific components have been shown to enhance outcomes for women with substance use disorders [24]. Therefore, it is important for future research to examine gender differences, as well as feedback on gender-specific elements of the program.

### Limitations

The small number of participants is a limitation in this study. Participants were recruited through a self-selection process which could be a limitation if participants who chose to partake in the survey were more positively biased than nonparticipants. Another limitation is that drinking to cope was not a criterion for eligibility, thus the intervention was not specifically tested on individuals who drink to cope. Individuals who drink to cope might have provided different data regarding acceptability and usefulness. Hence, future research should focus on testing the intervention in a target population with DTC. In addition, the sample was predominantly White, female, and college-educated. While this demographic distribution is representative of the community where the research was conducted, it limits the wider generalizability of the findings. Future research should seek to gain feedback on usability and satisfaction from a more diverse sample. Of note, recent research suggests alcohol use is increasing more among middle-aged and older adults than among younger drinkers [25], therefore the wide age range in the present sample could be an indication of the program’s appeal to this age group.

### Conclusions

In this QI study, we examined usability and satisfaction with a web-based Self-Coaching Plan that applies CBT and PPI principles featuring a novel framework of Vitality Type categories to engage clients in a structured self-development process to increase skills to manage stress and negative affect. Findings from this quality improvement study indicate that the Self-Coaching Plan was engaging as well as acceptable to participants, positively influencing their self-perception and behavior. Data from the custom questionnaire document that applying the framework of 4 Vitality Type patterns is an effective approach to empower individuals with information, strategies, and a plan to self-regulate. Taken together, results from this QI study warrant investigating if the Self-Coaching Plan’s approach to personalize assets-based self-management strategies targeting stress and negative affect, could help empower individuals with healthy habits to cope with stress instead of turning to alcohol.

## Abbreviations

CBT: cognitive behavioral therapy
DTC: drink to cope
HIPAA: Health Insurance Portability and Accountability Act
PPI: positive psychology intervention
QI: quality improvement
REDCap: Research Electronic Data Capture
SUS: System Usability Scale
